# Emergency umbilical hernia management: scoping review

**DOI:** 10.1093/bjsopen/zrae068

**Published:** 2024-06-20

**Authors:** Josephine Walshaw, Anna Kuligowska, Neil J Smart, Natalie S Blencowe, Matthew J Lee

**Affiliations:** Leeds Institute of Emergency General Surgery, St James’s University Hospital, Leeds, UK; Leeds Institute of Medical Research, St James’s University Hospital, University of Leeds, Leeds, UK; Leeds Institute of Emergency General Surgery, St James’s University Hospital, Leeds, UK; Department of Colorectal Surgery, Royal Devon and Exeter NHS Foundation Trust, Exeter, UK; Leeds Institute of Emergency General Surgery, St James’s University Hospital, Leeds, UK; Bristol Centre for Surgical Research, Population Health Sciences, University of Bristol, Bristol, UK; Leeds Institute of Emergency General Surgery, St James’s University Hospital, Leeds, UK; Institute for Applied Health Research, College of Medical and Dental Sciences, University of Birmingham, Birmingham, UK; Department of Trauma and Emergency General Surgery, University Hospitals Birmingham NHS Foundation Trust, Birmingham, UK

## Abstract

**Background:**

Umbilical hernias, while frequently asymptomatic, may become acutely symptomatic, strangulated or obstructed, and require emergency treatment. Robust evidence is required for high-quality care in this field. This scoping review aims to elucidate evidence gaps regarding emergency care of umbilical hernias.

**Methods:**

EMBASE, MEDLINE and CENTRAL databases were searched using a predefined strategy until November 2023. Primary research studies reporting on any aspect of emergency umbilical hernia care and published in the English language were eligible for inclusion. Studies were excluded where emergency umbilical hernia care was not the primary focus and subsets of relevant data were unable to be extracted. Two independent reviewers screened abstracts and full texts, resolving disagreements by consensus or a third reviewer. Data were charted according to core concepts addressed by each study and a narrative synthesis was performed.

**Results:**

Searches generated 534 abstracts, from which 32 full texts were assessed and 14 included in the final review. This encompassed 52 042 patients undergoing emergency umbilical hernia care. Most were retrospective cohort designs (11/14), split between single (6/14) and multicentre (8/14) with only one randomized trial. Most multicentre studies were from national databases (7/8). Themes arising included risk assessment (*n* = 4), timing of surgery (*n* = 4), investigations (*n* = 1), repair method (*n* = 8, four mesh *versus* suture; four laparoscopic *versus* open) and operative outcomes (*n* = 11). The most commonly reported outcomes were mortality (*n* = 9) and morbidity (*n* = 7) rates and length of hospital stay (*n* = 5). No studies included patient-reported outcomes specific to emergency umbilical hernia repair.

**Conclusion:**

This scoping review demonstrates the paucity of high-quality data for this condition. There is a need for randomized trials addressing all aspects of emergency umbilical hernia repair, with patient-reported outcomes.

## Teaser

Umbilical hernias, although frequently asymptomatic, may become acutely symptomatic, strangulated or obstructed, and require emergency treatment. Robust evidence is required for high-quality care in this field. This scoping review demonstrates the paucity of high-quality data and a need for randomized trials addressing all aspects of emergency umbilical hernia repair, with patient-reported outcomes.

## Background

An umbilical hernia is a common ventral hernia located at or near the umbilicus^1^. Although frequently benign and asymptomatic, these hernias can manifest acutely as symptomatic, strangulated, incarcerated or obstructed, thereby necessitating emergency intervention. The urgency of addressing these presentations is underscored by the potential risks associated with delayed surgery, including increased rates of major complications and prolonged hospital stay^2^.

Despite the clinical significance of emergency umbilical hernias, considerable diversity exists across the assessment and repair of acutely symptomatic hernias^3^, and the current landscape of evidence guiding their management remains notably underdeveloped. This variability highlights the need to understand the optimal strategies for management, incorporating elements such as risk assessment, perioperative care, timing of surgical intervention and selection of appropriate repair methods.

Robust evidence is required for high-quality care in this field, to inform clinical decisions and enhance patient outcomes. Therefore, this scoping review aims to provide a comprehensive overview of the current evidence for emergency care of umbilical hernias. By mapping the existing literature, it seeks to identify areas lacking evidence, thereby guiding future research priorities in emergency umbilical hernia care.

## Methods

This scoping review was conducted and reported according to the PRISMA extension for Scoping Reviews (PRISMA-ScR) guidance^4^ and Arksey and O’Malley’s five-stage scoping review process^5^.

### Eligibility criteria

Studies with a primary design addressing any aspect of emergency umbilical hernia repair in adult patients over the age of 18 were considered for inclusion. Studies were excluded where emergency umbilical hernia care was not the primary focus and subsets of relevant data were unable to be extracted. Studies focusing on emergency umbilical hernia repair in cirrhotic patients were also excluded. Reviews, case reports, editorials, conference abstracts and non-English language articles were excluded.

### Information sources

Systematic searches of EMBASE, MEDLINE and CENTRAL databases were performed using a predefined strategy on 10 October 2023. Reference lists of all included studies were manually reviewed to identify any additional relevant papers. In cases where full texts were inaccessible through conventional methods, authors were contacted via email. Searches were also performed on three trial databases (Clinicaltrials.gov, ICTRP and ISRCTN), using the term ‘umbilical hernia’. Studies were reviewed for status (open, recruiting, closed, etc.) and for the type of intervention being assessed.

### Search strategy

The search strategies were designed using relevant keywords and Medical Subject Heading (MeSH) terms, spanning from inception to November 2023 and limited to studies published in English (*Supplementary Data*  S1). Additional references were identified through hand-searches and from the grey literature.

### Selection of sources of evidence

Article screening was performed on Rayyan online software (rayyan.ai). Screening was carried out independently by two reviewers (JW and AK), with full texts of potentially relevant articles considered for inclusion. Disagreements between reviewers were resolved by consensus or with a third reviewer (MJL).

### Data items and charting process

Two reviewers (JW and AK) independently charted the data using bespoke Excel data extraction spreadsheets. Any disagreement between reviewers was resolved through consensus or with a third reviewer (MJL). Study descriptors were extracted including title, first author, year of publication, country of origin, study design, sample size, whether the study was single or multicentre and key findings from the paper. In order to group identified papers, key themes were determined from studies and used to create a framework. This was expected to correspond with the clinical pathway, addressing aspects such as diagnostics, treatment and outcomes.

### Synthesis of results

A narrative synthesis of the included studies was produced. A formal assessment of the risk of bias in the included studies was not performed in line with the methodology of scoping reviews^4^. Identification of gaps was undertaken by reviewing areas addressed by studies according to aspect of care addressed. Study findings were considered by the research team, and questions relevant to these evidence gaps were proposed.

## Results

**Fig. 1:**
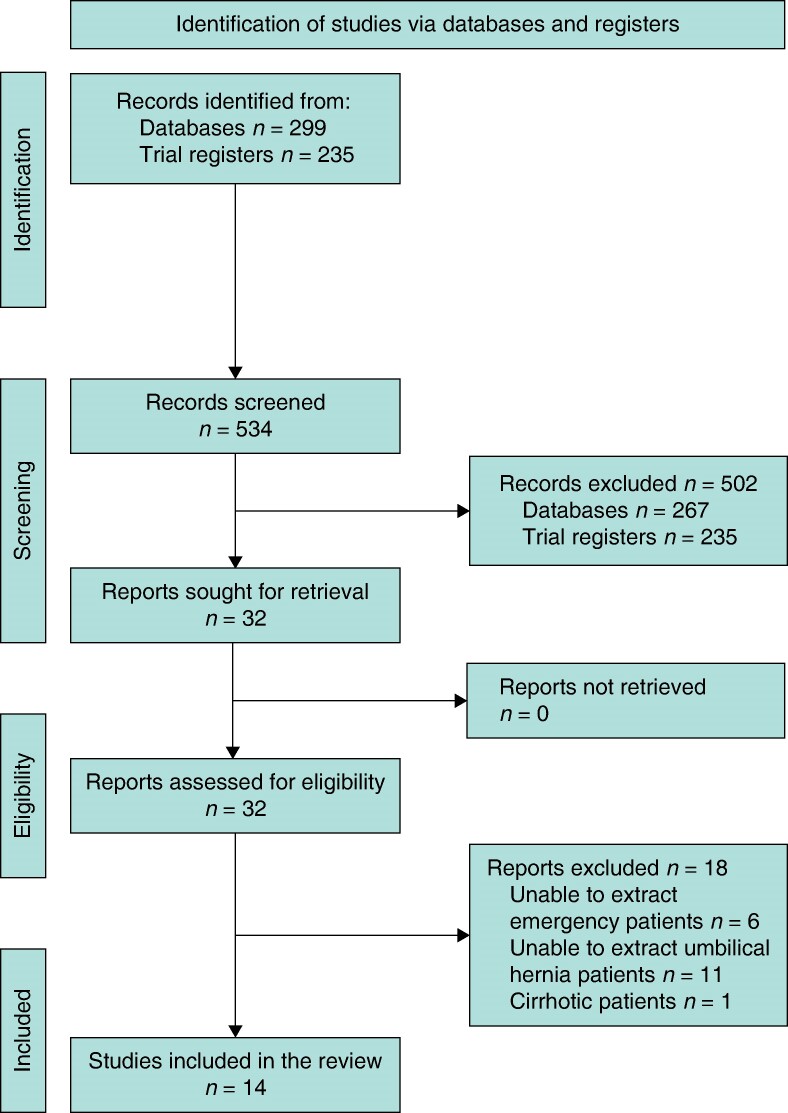
PRISMA flowchart

A total of 299 articles were identified for title and abstract screening, from which 32 full-text studies were reviewed (*Fig. 1*). Finally, 14 articles were eligible for inclusion in the review. A total of 235 articles were screened from the trial databases, none of which addressed emergency umbilical hernia repair.

### Study descriptors

The 14 studies encompassed a total of 565 025 patients, with 52 042 patients undergoing emergency umbilical hernia care (*Table 1*). Most were retrospective cohort designs (11/14), equally split between single (6/14) and multicentre (8/14) with only one RCT. Most multicentre studies were from national databases (7/8): the National Surgical Quality Improvement Program (NSQIP)^2,10,11^, the National Inpatient Sample (NIS)^12,13^, the Swedish National Patient Register^14^, and the Danish Hernia Database^15^.

**Table 1:** Study characteristics

First author	Year	Origin	Design	Single/multicentre	Total number of patients	Total number of emergency umbilical repairs	Maximum duration of follow-up
Ozkan^6^	2012	Turkey	Retrospective cohort	Single centre	2380	40	Not stated
Turan^7^	2022	Turkey	Retrospective cohort	Single centre	572	16	30 days
Emile^8^	2017	Egypt	Retrospective cohort	Single centre	122	109	Median 24 months (6–32 months)
Tomaoglu^9^	2021	Turkey	Retrospective cohort	Single centre	301	58	Mean 18.2 months (1–42 months)
Leeds^2^	2020	USA	Retrospective cohort	Multicentre (NSQIP)	76 364	15 426	30 days
Savitch^10^	2016	USA	Retrospective cohort	Multicentre (NSQIP)	112 074	5954	30 days
Bohnen^11^	2016	USA	Retrospective cohort	Multicentre (NSQIP)	170 131	822	Not stated
Colavita^12^	2013	USA	Retrospective cohort	Multicentre (NIS)	18 223	3258	No follow up
Patel^13^	2022	USA	Retrospective cohort	Multicentre (NIS)	21 242	21 242	Not stated
Ali^14^	2023	Sweden	Retrospective cohort	Multicentre (Swedish Register)	152 192	4214	No follow up
Helgstrand^15^	2013	Denmark	Retrospective cohort	Multicentre (Danish database)	10 976	733	30 days
Bouliaris^16^	2022	Greece	Prospective cohort	Single centre	86	15	15 days and 30 days
Proctor^17^	2022	UK	Prospective cohort	Multicentre	273	113	30 days and 90 days
Abdel-Baki^18^	2007	Egypt	RCT	Single centre	42	42	Mean 16 months (6–24 months)

NSQIP, National Surgical Quality Improvement Program; NIS, National Inpatient Sample.

**Fig. 2:**
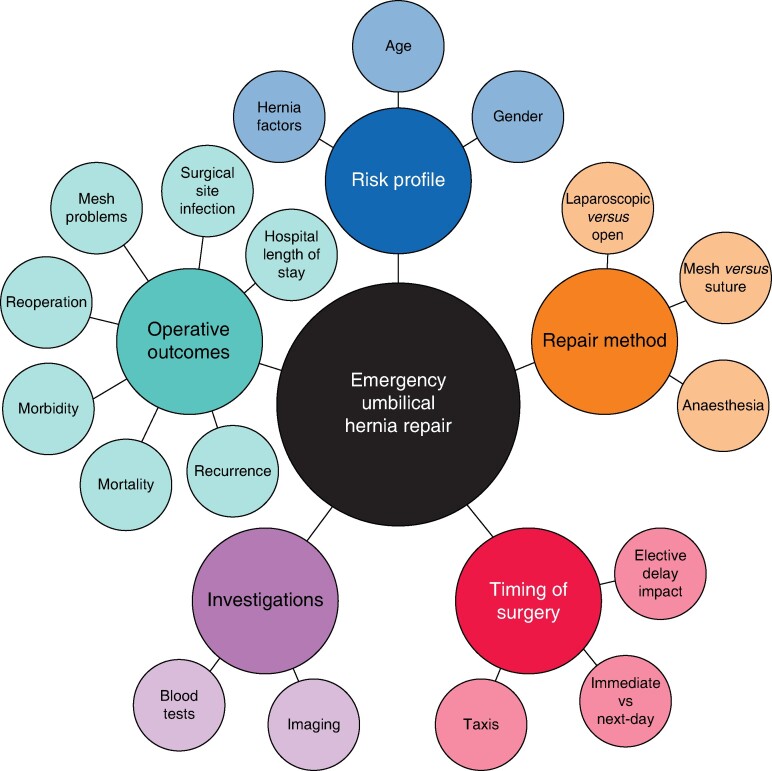
Data mapping to key emergency umbilical hernia repair themes

Study results were classified into a framework encompassing five overarching themes: risk profile, timing of surgery, investigations, repair methods and operative outcomes. Studies spanning multiple themes were appropriately included in all relevant categories. A concept map was generated to visualize the evidence and demonstrate any gaps in the evidence (*Fig. 2*).

### Risk profile

The risk profile for emergency umbilical hernia repair was reported in four studies^6,11,13,15^, all of which were retrospective cohorts. Among them, three were multicentre studies using national databases^11,13,15^ and one was a single-centre study^6^. Notably, these analyses described a heightened risk of major morbidity and mortality associated with emergency *versus* non-emergency repair^11,15^. Factors explored in relation to outcomes included gender, age, hernia characteristics and treatment payment method.

Regarding gender, one study reported umbilical hernias were more frequently seen in women^6^. Female gender emerged as an independent risk factor for emergency umbilical hernia repair in one study^15^, yet in another study was noted to be a protective factor for mortality^13^. In retrospective database analysis, advancing age was described as a risk factor for both requiring an emergency repair^15^ and experiencing a higher mortality rate regardless of whether the patient underwent surgery following emergent admission^13^. Within the adult population (age 18–64 years), the main risk factors identified as being associated with mortality in one study were age and gangrene, a pattern that persisted in the elderly population (age 65 and over) with the addition of frailty^13^. Additional risk factors reported to increase the risk of emergency umbilical repair in another study included having a hernia defect between 2 and 7 cm and the hernia being primary rather than recurrent^15^. A retrospective exploration of the impact of treatment payment methods in a United States cohort indicated a higher likelihood of mortality among adult operative patients with Medicare, Medicaid or self-paying status compared to those with private insurance^13^.

### Timing of surgery

Four studies described the impact of the timing of emergency umbilical hernia repair^2,7,14,16^. Two were retrospective cohort studies from national databases^2,14^ and two were small single-centre studies^7,16^. A study of the NSQIP database found that, despite overall increased odds of a major complication with next-day surgery for all emergency hernia presentations, multivariable logistic regression for next-day umbilical hernia surgery was associated with decreased odds of a major complication compared to surgery delayed more than one day^2^.

Three studies explored the impact of the coronavirus disease 2019 (COVID-19) pandemic on hernia repair^7,14,16^. Findings regarding the relationship between reduced elective hernia repair and the number of emergency presentations were contradictory. One study reported no correlation between the number of elective and emergency umbilical hernia repairs performed^14^ whereas another stated there was a two-fold increase in the number of emergency ventral (including both umbilical and incisional) hernia repairs compared to the pre-pandemic period^7^. During the pandemic, one study investigated the manual reduction, or taxis, of incarcerated umbilical hernias^16^. Of 15 patients, 9 had successful reduction (with 4 going on to have elective repair) and 6 had unsuccessful reduction necessitating emergency surgery.

### Investigations

One multicentre prospective cohort discussed investigations conducted prior to emergency umbilical hernia repair^17^. It described the utilization of imaging modalities, revealing that 33.7% underwent abdominal radiography, 5.0% had ultrasound imaging and 47.5% had a CT scan^17^. Additionally, it provided information on the median (interquartile range) white blood cell count (8.4, 7.0–11.2 × 10^9^/l), C-reactive protein levels (5, 2–10 mmol/l) and patients with acute kidney injury (2.0%)^17^.

### Repair method

Eight studies detailed repair methods employed for emergency umbilical hernias^6,8–10,12,15,17,18^. Among them, four studies explored the use of prosthetic mesh repair *versus* suture repair^8,9,17,18^—two retrospective single-centre studies^8,9^, one multicentre prospective cohort^17^ and one small RCT^18^. In cohort studies, there was reported variation in the number of patients who underwent mesh *versus* suture repair. In these, one study found no significant difference in the number of patients who received mesh *versus* suture repair^8^, one study observed a greater proportion of patients who underwent mesh repair^9^, whereas another study reported a greater number who underwent suture repair^17^. Additionally, one study also discerned no variation in the rate of mesh use between elective and emergency repairs^15^.

The impact of repair type on operation time exhibited variability, with one study reporting prosthetic mesh repair was associated with a longer mean operative time^18^ whereas another study found no significant difference in operative time concerning both hernia type and repair type^9^. There was also variation reported in hospital length of stay (HLOS), with one study indicating no differences between repair types^18^ and another reporting significantly longer HLOS for umbilical hernia repairs in general, and primary hernia repairs without specific breakdown by hernia type^9^.

One study explored risk factors for surgical site infection (SSI) including demographic and operative factors; although overall mesh repair was not found to be a significant risk factor for SSI in this study (7.5% for mesh *versus* 5.3% for suture), the analysis grouped hernia types together, impeding specific reporting^8^. Regarding hernia recurrence, one study evaluating emergency repair of multiple hernia types reported that all early hernia recurrence in their study was observed in umbilical hernias, 83.3% of which were suture repair^17^. In the longer term, all studies highlighted a higher rate of hernia recurrence in suture repair^8,9,17,18^. There were no reported long-term complications related to the presence of mesh reported in an RCT of 42 patients having emergency umbilical hernia surgery^18^.

Four studies discussed laparoscopic *versus* open emergency repair of umbilical hernias^10,12,15,17^, three multicentre studies from national databases^10,12,15^ and one multicentre prospective cohort^17^. Open repair was reported to be more commonly utilized in the emergency setting across all studies, with one study identifying non-emergent admission as an independent predictor of undergoing a laparoscopic repair^12^. The conversion rate of laparoscopic to open repair was reported to be 1% in one study^17^.

Two studies reported on the type of anaesthesia used during emergency umbilical hernia repair^6,17^. General anaesthesia was the most common mode of anaesthesia, utilized in 100% of cases in one study^6^ and 96% in the other^17^, with spinal anaesthesia (2%) and local anaesthesia (1%) being less commonly reported modes. One study reported on the grade of operating surgeon, with almost half of the repairs being performed by a consultant surgeon (49%)^17^.

### Operative outcomes

**Fig. 3:**
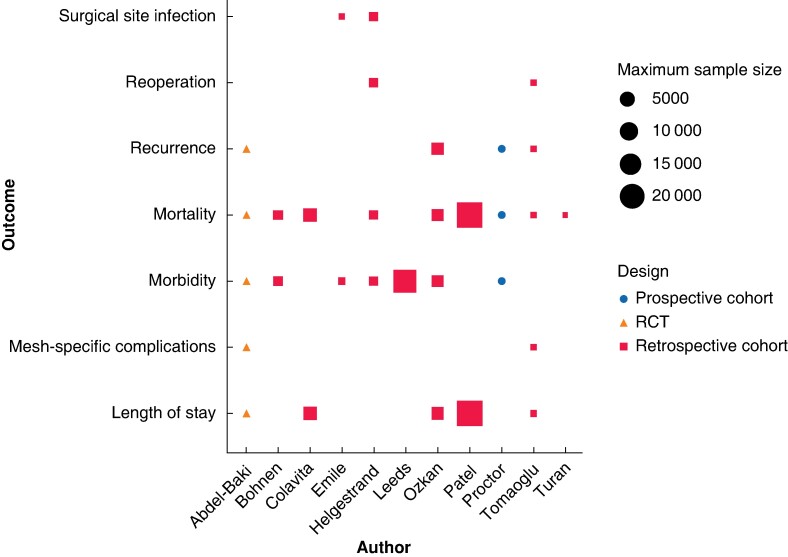
Reported operative outcomes for emergency umbilical hernia repair, according to study design and population size

The reported outcomes associated with emergency umbilical hernia repair varied across the reviewed studies (*Fig. 3*). The most commonly reported outcomes were mortality (*n* = 9) and morbidity (*n* = 7) rates and length of hospital stay (*n* = 5), and there was no one outcome consistently reported across all studies. Notably, three studies did not provide specific operative outcomes for emergency umbilical hernia repair; two studies combined operative outcome reporting of hernia types^10,16^ and one study focused on the relationship between reduced or delayed elective surgery and increased emergent repairs, without detailing operative outcomes^14^.

Regarding intraoperative outcomes, three studies indicated whether a bowel resection was performed during the operation^9,15,18^. The rate of bowel resection was reported to be significantly higher in emergency umbilical hernia repairs compared to elective repairs at 2.5% *versus* 0%^15^. A subgroup analysis comparing groin hernias (femoral and inguinal) against ventral hernias (incisional and umbilical) found no significant difference between the two groups in patients who underwent bowel resection or not; however, no separate data for emergency umbilical hernia repair are reported^9^. No studies were identified that assessed patient outcomes for laparoscopic *versus* open emergency repair of umbilical hernias.

In terms of postoperative recovery, HLOS for emergency umbilical hernias was variable and reported in five papers^6,9,12,13,18^. A study comparing adult (age 18–64 years) and elderly patient (age 65 and over) populations found that elderly patients who had emergency umbilical hernia repair had a longer average HLOS than adult patients (5.93 ± 6.45 days *versus* 3.75 ± 5.67 days), with higher HLOS for deceased patients in both age groups^13^. Notably, this study also reported that HLOS was a risk factor for mortality in adult patients admitted emergently who did not have surgery, raising the mortality rate by 7.7% for each additional day^13^. Postoperative morbidity was examined across seven studies^2,6,8,11,15,17,18^, with specific attention to SSI in three papers^8,15,18^. Two studies found no difference in SSI rates in mesh repair^8,18^, and one study reported one of the three patients who had mesh implanted after bowel resection developed wound infection^9^. Furthermore, in the subset of studies exploring mesh repair for emergency umbilical hernia repair, only two of the five reported on mesh-related complications^9,18^.

Postoperative follow-up was reported in nine studies^2,7–10,15–18^, ranging from 30 days to 42 months. Hernia recurrence rates were described in four studies^6,9,17,18^—this was reported as more common in suture repair in both the early^17^ and late^9,18^ postoperative period. Reoperation rates following emergency umbilical hernia repair were reported in two studies, ranging between 2.0%^9^ and 3.8%^15^. Mortality was the most commonly included operative outcome, being described in nine studies^6,7,9,11–13,15,17,18^. Again, mortality rates for emergency umbilical hernia repair were variable, reported between 0%^6,18^ and 5.9%^9^. Although one study assessed the quality of life for all patients who presented emergently with any kind of hernia^17^, no studies reported on the quality of life specific to emergency umbilical hernia repair.

### Gaps assessment

The evidence was summarized into a framework based on *Fig. 2*. Uncertainties were identified through discussion between reviewers and identified gaps are presented in *Table 2*. These demonstrate a high level of uncertainty in all aspects of care of this condition.

**Table 2:** Identified evidence gaps in emergency umbilical hernia repair

Themes	Subthemes	Frequency (number)	Identified gaps
Risk profile (*n* = 4)	Age	2	What are the characteristics of a hernia that may predict the requirement for emergency surgery? Who is at risk of poor outcomes after emergency surgery?
	Gender	3	
	Hernia factors	2	
Timing of surgery (*n* = 4)	Elective delay impact	2	In what context is taxis a safe intervention for umbilical hernias? How long can people wait for a repair without requiring emergency surgery? When is it safe to defer surgery until the next day?
	Immediate *versus* next-day	1	
	Taxis	1	
Investigations (*n* = 1)	Imaging	1	What imaging is required to inform decision-making? What blood tests can predict outcomes from surgery?
	Blood tests	1	
Repair method (*n* = 8)	Laparoscopic *versus* open	4	Does laparoscopic or open repair offer better outcomes? Does a mesh or suture-based repair offer better short- and long-term outcomes? Is a general anaesthetic required for a repair?
	Mesh *versus* suture	4	
	Anaesthesia	2	
Operative outcomes (*n* = 11)	Hospital length of stay	5	What outcomes are the most important for patients and clinicians in emergency hernia surgery? When should outcomes be measured? How can we optimize patient outcomes?
	Surgical site infection	3	
	Mesh problems	2	
	Reoperation	2	
	Recurrence	4	
	Morbidity	7	
	Mortality	9	
Unaddressed areas	How should patients be optimized before emergency repair? Should antibiotics be used routinely in emergency repair? What postoperative care practices positively influence patient outcomes?		

## Discussion

This scoping review provides a comprehensive overview of the existing literature on emergency umbilical hernia repair, summarizing available evidence relating to risk profiles, timing of surgery, investigations, repair methods and outcomes. Key in this analysis is the demonstration of a paucity of high-level evidence regarding most aspects of practice.

The main findings of this review suggest a heightened risk of major morbidity and mortality within emergency repair, with notable variations in risk factors such as age, gender and hernia characteristics. The timing of surgery, impacted by factors such as the COVID-19 pandemic, showcased variable findings, reflecting the dynamic nature of the healthcare setting. Preoperative investigations and repair methods also demonstrated diversity, emphasizing the need for a larger evidence base and standardization. The reported outcomes demonstrate variability in intraoperative procedures, postoperative recovery and associated morbidity and mortality, underscoring the complexity and heterogeneity of emergency umbilical hernia outcomes.

There is limited evidence on mesh utilization in emergency umbilical hernia repair, particularly following intestinal ischaemia and resection. This is reflected in recent surveys and cohort studies, which found variation in the type of repair offered for acute umbilical hernia^3,17^. The European Hernia Society and American Hernia Society guidelines for the treatment of umbilical and epigastric hernias provide only one weak recommendation focused on emergency repair, stating there is low-level evidence to suggest non-resorbable mesh is safe in clean or clean-contaminated repair^19^. The current review indicates that the up-to-date evidence for emergency umbilical hernia repair with mesh was not linked with higher SSI rates^8,18^. Furthermore, no long-term complications were identified related to the presence of mesh^9,18^ and reduced recurrence rates compared to suture repair^9,17,18^. These findings contribute to the ongoing discourse surrounding the effectiveness and safety of mesh use and emphasize the need for more targeted investigation to inform evidence-based practices.

The omission and variation of operative outcome data specific to emergency umbilical hernia repair was found to be a significant limitation in current literature. This is also noted in a previous systematic review of outcome reporting in incisional hernia surgery, which found significant heterogeneity, with variation in outcome assessment and definitions^20^. There were no studies in this review directly comparing umbilical hernia outcomes for laparoscopic and open emergency repair. There is evidence to suggest that, despite its relatively low utilization in the emergency setting, laparoscopic repair is associated with decreased HLOS and lower rates of wound-related morbidity^21,22^. Although these findings provide valuable insight into the potential benefits of laparoscopic approaches, the lack of outcome data for emergency umbilical hernia repair underscores the need for further research.

Moreover, this review highlights the absence of studies addressing the quality of life specifically related to emergency umbilical hernia repair. One included study did identify improvements at 30 and 90 days using the EuroQol EQ-5D-5L tool, with no significant difference in those who underwent mesh *versus* suture repair; however, there were no hernia-type specific quality of life outcomes reported^17^. This again has been noted in the literature, with patient-reported outcomes reported in 34% of studies assessing incisional hernia repair^20^. The standardization of outcome reporting for emergency hernia surgery and a concerted effort to address existing gaps in the literature should be a priority.

A notable challenge in the existing literature is the combined reporting of hernia types, impeding analysis of specificities related to emergency umbilical hernia repairs. This lack of granularity hinders the ability to draw targeted conclusions about the specific risk factors, outcomes, and management strategies unique to umbilical hernias. Where research includes various hernia types, researchers should ensure disaggregation of hernia types to allow for a more accurate understanding of the unique characteristics and challenges posed by each. Additionally, the preponderance of retrospective, multicentre studies relying on national databases raises concerns that the studies may not adequately capture the intricacies of emergency presentations and specific nuances related to procedural interventions.

Undertaking RCTs in an emergency surgical setting is both practically and methodologically challenging, with factors including difficulties with recruitment, lack of personal clinical equipoise, and issues surrounding informed consent^23,24^. The scarcity of RCTs in the current body of literature hinders the establishment of clear cause-and-effect relationships between interventions and outcomes in the context of emergency umbilical hernia repair. Future research endeavours should aim for more robust study designs, standardized outcome measures and a focus on patient-centred outcomes to further advance the understanding and management of emergency umbilical hernia repair. Such a trial might randomize comparators such as repair materials, drain use, antibiotic use, taxis *versus* surgery and wound closure.

This review focuses on mapping the current literature and incorporating diverse evidence sources rather than synthesizing quantitative evidence. Although this approach is suitable to capture a holistic understanding of this topic, it may limit the ability to draw definite conclusions and provide quantitative insights into the effectiveness of specific interventions. Another limitation of the scoping review methodology is the lack of study quality assessment, which may introduce studies of varying methodological quality into the review, impacting the overall robustness of the findings.

This review illuminates the evidence gaps and signals the importance of future studies specifically focusing on emergency interventions for umbilical hernias. This is an area of need. The 2017 update of the World Society of Emergency Surgery (WSES) guidelines for emergency repair of complicated abdominal wall hernias, although informative for a broader context, does not provide specific detailed guidance on the emergency management of umbilical hernias^25^. This limited guidance is reflected across other guideline documents. Consequently, this review has implications for clinicians and researchers. The gaps analysis highlights a relative paucity of literature to guide the management of this type of hernia presentation. Many of the gaps are easily addressable in well-designed prospective studies. There is a clear need for policymakers, funders, and specialist societies to support the development and delivery of trials in this field.

## Supplementary Material

zrae068_Supplementary_Data

## Data Availability

No new data were generated or analyzed in support of this research.

## Supplementary material

Supplementary  material is available at *BJS Open* online.
