# Supplementary material for: Emergency umbilical hernia management: scoping review
Source: BJS Open. 2024 Jun 20;8(3):zrae068. doi: 10.1093/bjsopen/zrae068 (PMC11186979; doi:10.1093/bjsopen/zrae068)
Supplement: zrae068_Supplementary_Data [file zrae068_supplementary_data.docx]

**Supplementary data 1:** Medline Search Strategy

| 1 | exp hernia, umbilical/ |
| --- | --- |
| 2 | exp hernia, ventral/ |
| 3 | ((Umbili* or paraumb* or ventr*) and herni*).mp |
| 4 | 1 or 2 or 3 |
| 5 | exp Acute Disease/ |
| 6 | exp Emergencies/ |
| 7 | (acute or emergen* or urgent* or strangu* or incarc* or obstru*).mp |
| 8 | 5 or 6 or 7 |
| 9 | 3 and 8 |
| 10 | exp animals/ not humans.sh |
| 11 | 9 not 10 |
| 12 | randomized controlled trial.pt |
| 13 | controlled clinical trial.pt |
| 14 | randomi?ed.ab |
| 15 | placebo.ab |
| 16 | randomly.ab |
| 17 | trial.ab |
| 18 | groups.ab |
| 19 | 12 or 13 or 14 or 15 or 16 or 17 or 18 |
| 20 | 11 and 19 |
| 21 | brain.mp |
| 22 | 20 not 21 |
